# Supplementary material for: Silver Nanoparticles Induce Neutrophil Extracellular Traps Via Activation of PAD and Neutrophil Elastase
Source: Biomolecules. 2021 Feb 19;11(2):317. doi: 10.3390/biom11020317 (PMC7922014; doi:10.3390/biom11020317)
Supplement: Supplementary file 1 [file biomolecules-11-00317-s001.pdf]

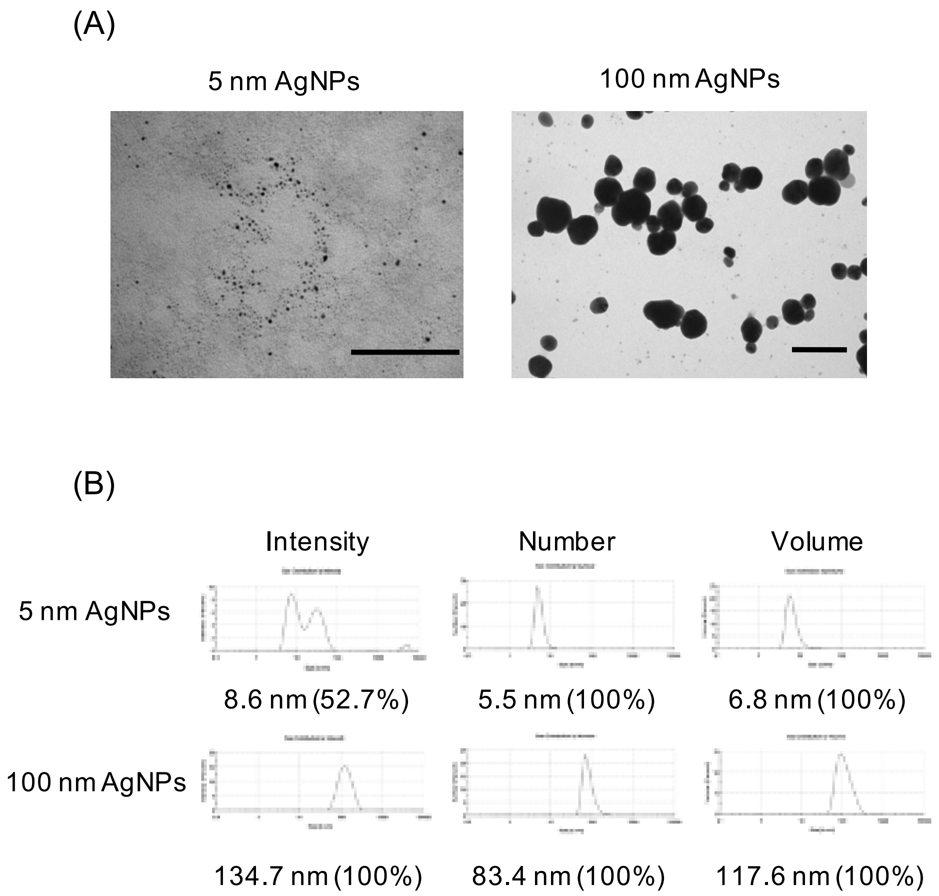


**Figure S1. Characteristics of AgNPs.** (**A**) TEM images of AgNPs show that 5 nm and 100 nm nanoparticles are relatively uniform in size and well distributed. Scale bar: 200 nm. (**B**) DLS analysis by number reveals mean sizes are 5.5 nm for 5 nm AgNPs and 83.4 nm for 100 nm AgNPs. For DLS analysis, AgNPs were dispersed in RPMI 1640 containing 1% FBS.

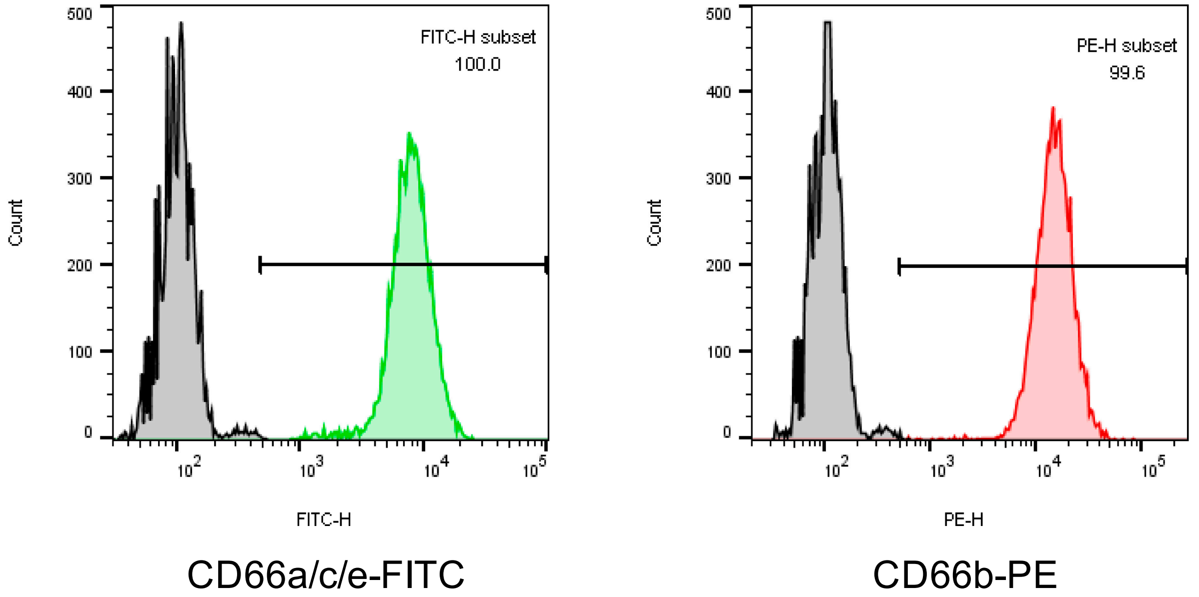


**Figure S2. Purity of isolated neutrophils.** Isolated human neutrophils were stained CD66a/c/e and CD66 b antibody and analyzed by flow cytometry. The purity of neutrophils more than 95%.

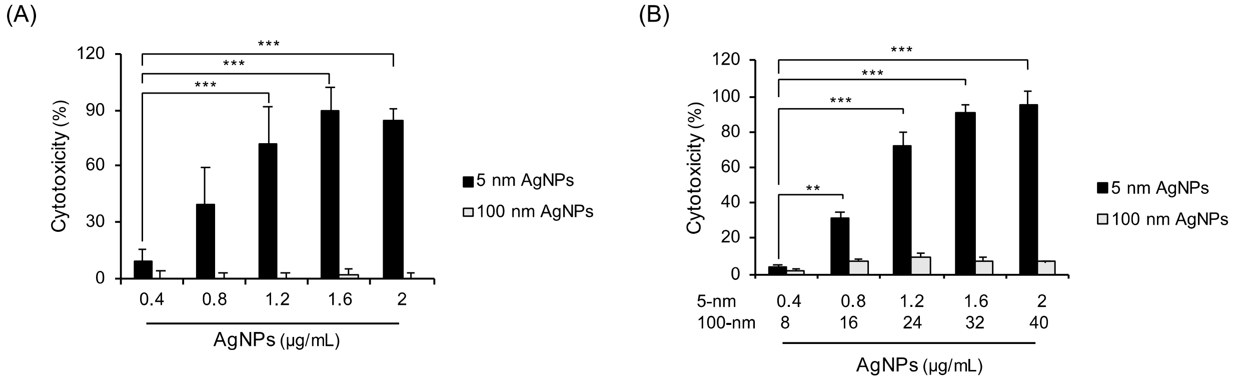


**Figure S3. Cytotoxicity of AgNPs in human neutrophils.** Hunan neutrophils were treated with AgNPs for 4 h, and cytotoxicity was measured with the LDH assay. (**A**) Cytotoxicity was measured at the same concentration for both 5 nm and 100 nm AgNPs. The $LD_{50}$ of the 5 nm AgNPs was 0.93 μg/mL. (**B**) Cytotoxicity was measured at different concentrations that provided the same surface area. One-way ANOVA was used to determine the significance (**$p < 0.01$, ***$p < 0.001$).

Table 1. PCR primers used in this study.

| Primer | Sequnce (5'-3') |
|---|---|
| Mitochondrial genes | |
| *MT-ND1* | Forward: GCA TTC CTA ATG CTT ACC GAA C<br>Reverse: AAG GGT GGA GAG GTT AAA GGA G |
| *MT-CYB* | Forward: GAC CCA GAC AAT TAT ACC CTA GCC<br>Reverse: CCT CCG ATT CAG GTT AGA ATG AGG |
| Nuclear genes | |
| *GAPDH* | Forward: AGG GCC CTG ACA ACT CTT TT<br>Reverse: AGG GGT CTA CAT GGC AAC TG |
| *FAS* | Forward: TCA CCA CTA TTG CTG GAG TCA T<br>Reverse: TAA ACA TCC TTG GAG GCA GAA T |
